# Outpatient Oral Neuropathic Pain Management with Photobiomodulation Therapy: A Prospective Analgesic Pharmacotherapy-Paralleled Feasibility Trial

**DOI:** 10.3390/antiox11030533

**Published:** 2022-03-10

**Authors:** Reem Hanna, René Jean Bensadoun, Seppe Vander Beken, Patricia Burton, James Carroll, Stefano Benedicenti

**Affiliations:** 1 Department of Oral Surgery, Dental Institute, King’s College Hospital NHS Foundation Trust, London SE5 9RS, UK; 2 Department of Surgical Sciences and Integrated Diagnostics, Laser Therapy Centre, University of Genoa, Viale Benedetto XV,6, 16132 Genoa, Italy; stefano.benedicenti@unige.it; 3 Department of Radiology Oncology, Centre De Haute Energie, 10 Boulevard Pasteur, 06000 Nice, France; renejean.bensadoun@che-nice.com; 4 Bredent Medical GmbH & Co., Gewerbegebiet Gartenäcker, Weißenhorner Str. 2, 89250 Senden, Germany; seppe_vb@hotmail.com; 5 Thor Photomedicine Ltd., Water Meadow, Chesham HP5 1LF, UK; Patricia.burton@thorlaser.com (P.B.); james.carroll@thorlaser.com (J.C.)

**Keywords:** burning mouth syndrome, EQ-5D-5L, functional problems, IMMPACT, neuropathic pain, oral iatrogenic neuropathy, pharmacotherapy, photobiomodulation therapy, psychological dysfunction, PROMs, standard analgesic

## Abstract

Neuropathic pain (NP) can be challenging to treat effectively as analgesic pharmacotherapy (MED) can reduce pain, but the majority of patients do not experience complete pain relief. Our pilot approach is to assess the feasibility and efficacy of an evidence-based photobiomodulation (PBM) intervention protocol. This would be as an alternative to paralleled standard analgesic MED for modulating NP intensity-related physical function and quality of life (QoL) prospectively in a mixed neurological primary burning mouth syndrome and oral iatrogenic neuropathy study population (*n* = 28). The study group assignments and outcome evaluation strategy/location depended on the individual patient preferences and convenience rather than on randomisation. Our prospective parallel study aimed to evaluate the possible pre/post-benefit of PBM and to allow for a first qualitative comparison with MED, various patient-reported outcome measures (PROMs) based on Initiative on Methods, Measurement, and Pain Assessment in Clinical Trials (IMMPACT-II) were used for up to a nine-month follow-up period in both intervention groups (PBM and MED). The PBM protocol applied to the PBM group was as follows: λ810 nm, 200 mW, 0.088 cm^2^, 30 s/point, 9 trigger and affected points, twice a week for five consecutive weeks, whereas the MED protocol followed the National Institute of Clinical Excellence (NICE) guidelines. Our results showed that despite the severe and persistent nature of the symptoms of 57.50 ± 47.93 months at baseline in the PBM group, a notably rapid reduction in PIS_max_ on VAS from 7.6 at baseline (T0) to 3.9 at one-month post-treatment (T3) could be achieved. On the other hand, mean PIS_max_ was only reduced from 8.2 at baseline to 6.8 at T3 in the MED group. Our positive PBM findings furthermore support more patients’ benefits in improving QoL and functional activities, which were considerably impaired by NP such as: *eating, drinking and tasting*, whereas the analgesic medication regimens did not. No adverse events were observed in both groups. To the best knowledge of the authors, our study is the first to investigate PBM efficacy as a monotherapy compared to the gold standard analgesic pharmacotherapy. Our positive data proves statistically significant improvements in patient self-reported NP, functionality, psychological profile and QoL at mid- and end-treatment, as well as throughout the follow-up time points (one, three, six and nine months) and sustained up to nine months in the PBM group, compared to the MED group. Our study, for the first time, proves the efficacy and safety of PBM as a potent analgesic in oral NP and as a valid alternative to the gold standard pharmacotherapy approach. Furthermore, we observed long-term pain relief and functional benefits that indicate that PBM modulates NP pathology in a pro-regenerative manner, presumably via antioxidant mechanisms.

## 1. Introduction

Neuropathic pain (NP) is an altered sensation in the orofacial region [1] due to a lesion or a disease of the somatosensory nervous system, which interferes with every social interaction and causes a significant loss of function [2,3]. This can have negative effects on a patient’s self-image and quality of life (QoL), resulting in a significant negative psychological effect [4].

Trigeminal nerve (TN) injury following oral surgical interventions is one of the clinical manifestations of oral NP [5]. Equally, the International Association of Study Pain (IASP) has identified burning mouth syndrome (BMS), as a “distinctive neuropathic entity” [6] commonly affecting the anterior two-thirds of the tongue, where the peripheral nerves are distributed [7,8]. In 2018, the International Headache Society (HIS) has given further specifications of idiopathic or primary BMS for diagnostic criteria purposes and defined it as “an intraoral burning or dysaesthetic sensation, recurring daily for more than two hours per day over more than three months, without evident causative lesions on clinical examination and investigation” [9]. Although the precise etiopathogenesis remains unclear, neurophysiological studies have suggested that BMS is neuro-pathogenic in nature, indicating a dysfunction at the peripheral and central reflex arc paths and the processing of cortical excitation [10,11]. The most accepted theory explains the partial or total loss of chorda tympani nerve function, resulting in pain along TN pathways, as both taste and pain systems are regulated by central nervous system interneurons [12,13]. Subsequently, a cascade of the following molecular and chemical changes occurs: an increase in reactive oxygen species (ROS), adenosine triphosphate (ATP) production imbalance, cytosolic Ca^+2^ imbalance mechanism, which can lead to a failure in the Na^+^/K^+^ ATPase and in primary sensory neurons [14]. This may contribute to NP’s ectopic activity characteristic [15,16], which have both been previously implicated in NP pathogenesis [17,18]. In this context, BMS is a trigeminal small-fibre sensory neuropathy because of axonal degeneration and a decrease of epithelial nerve fibres [8,19].

Studies showed that pharmacological and non-pharmacological strategies in NP management are challenging [20,21,22,23]. It is noteworthy that some systematic medications are effective in the short term but can be associated with major side effects, threatening the large-scale and long-term applicability [24]. Hence, photobiomodulation (PBM) therapy could be a promising alternative treatment modality. The photonic energy in PBM is presumably absorbed within intracellular mitochondria by cytochrome C oxidase, where it binds to nitric oxide (NO) [25,26] and decouples it in a stressed cell, resulting in measurable ROS reduction and an ATP production upswing [27]. In nerve cells, this correlates with reduced pain stimulus conductance based on a plethora of postulated molecular and cellular modes of action, as well as its obvious experimentally observed analgesic effects [28,29,30,31,32].

**Figure 1:**
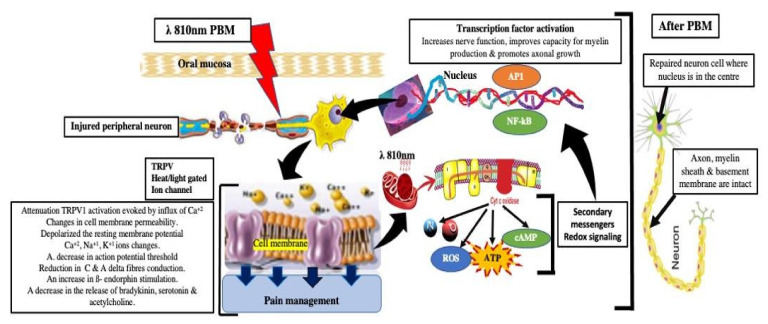
Schematic description of the PBM effects on injured peripheral neurons via its primary, secondary and tertiary activities. Several signaling pathways are induced by PBM irradiation, in reducing neuropathic pain and pro-inflammatory cytokines and neurotransmitter mediators. Abbreviations: PBM: photobiomodulation; TRPV1: transient receptor potential cation channel subfamily V member 1; Ca^+2^: calcium ion; K^+1^: potassium ion; Na^+1^: sodium ion; cyt c oxidase: cytostome c oxidase; CAMP: cyclic adenosine monophosphate, ROS: reactive oxygen species; ATP: adenosine triphosphate; NO: nitric oxide; AP1: activation protein1; NF-kB: nuclear factor-kappa (Supplementary File S2).

The analgesic effect of PBM, however, is primarily due to an increased release of β-endorphin, serotonin and enkephalins (natural endogenous opioid neuropeptides), acting to attenuate substance P release, bradykinin, histamine and prostaglandin E2 secretions that inhibit the afferent pain fibres [33]. By causing reversible changes in membrane permeability, the therapeutically applied photons furthermore stimulate cell activity and proliferation, while decreasing C and A delta fibre activity [13,34]. At the supra-cellular level, PBM has been correlated with increased microcirculation, modulation of neurotransmission and improved nerve regeneration, numbers of proliferating fibroblasts and macrophages and a decrease in inflammatory cytokines, amongst others demonstrated by tumour necrosis factor-alpha (TNF-α) and interleukin-1beta (IL-1β) levels [35,36,37]. PBM alters the nerve conduction and excitation in peripheral neurons by its action on the Na+/K+ pump [38], resulting in noxious stimuli reduction, through its effects on transient receptor potential cation channel subfamily V member 1 (TRPV1) and nerve growth factor (NGF) signaling blockers, decreasing their expressions (blockage of inflammatory thermal hyperalgesia) [39]. Nevertheless, the physiological mechanisms underlying pain reduction after PBM remain unclear [40]. Figure 1 illustrates the aforementioned mechanisms of action of PBM and signaling pathways in pain management.

Although several clinical RCT studies have already documented and provided evidence of PBM effectiveness in reducing NP intensity, improving the functionality and QoL [35,41,42,43,44,45,46,47,48,49,50,51], the most recent systematic reviews and meta-analyses [52,53,54] highlighted a high degree of heterogeneity in these studies with respect to the methodology and laser protocols used.

Applicability studies that evaluate the acceptance and feasibility of a well-defined PBM procedure in relation to its efficacy in pain management and improving the functionality and QoL in patients with chronic orofacial pain are still missing. Such studies are needed in order to establish standardised PBM for daily practice as a possible alternative or adjuvant therapy to analgesic medication in outpatient care. 

Here, we embraced a prospective pilot approach to assess the feasibility and efficacy of an evidence-based PBM intervention protocol as an alternative to paralleled standard analgesic pharmacotherapy for modulating NP intensity, related physical function and QoL in a mixed primary BMS and oral iatrogenic neuropathy (OIN) study population. To this end, study group assignments and outcome evaluation strategy/location depended on the individual patient preferences and convenience rather than on randomisation. It aimed to evaluate the possible pre/post-benefit of PBM as well as to allow for a first qualitative comparison with the standard care analgesic medication, various patient-reported outcome measures (PROMs) were used in this study for up to a nine-month follow-up period in both intervention groups. 

## 2. Materials and Methods

### 2.1. Study Design

A comparative experimental non-randomised intervention clinical study was conducted to evaluate the efficacy of laser-PBM (PBM group), compared to the pharmacotherapy (MED group) in NP management. The blinding strategies in our study were as follows: blinding of data collectors and outcome adjudicators. The performer of the laser treatment is a clinician with a wealth of experience in the fields of laser therapy and pharmacotherapy of NP. The New Procedure Committee (NPC) at King’s College Hospital NHS Foundation Trust, London, UK approved the study in utilising laser-PBM (λ810 nm) in treating chronic NP for BMS and oral iatrogenic nerve injury (OINI) cohort. Informed written consent was obtained from all the recruited participants, after a full explanation of the proposed treatments and alternative treatment modalities, in terms of advantages and drawbacks. The investigations were performed according to the Declaration of Helsinki on Biomedical Studies Involving Human Subjects. The study protocol was designed using the recommendations of the Consolidated Standards of Reporting Trials (CONSORT) extension statement for pilot and feasibility trials checklist (CONSORT 2010 Statement: extension to randomised pilot and feasibility trials [55] and followed their checklist (Supplementary File S1).

The primary objectives were to assess pain modulation and patients’ acceptability of PBM, related to adverse events and treatment compliance, during the intervention period and up to nine months of follow-up, as compared to pharmacotherapy that represents the standard-of-care reference. The secondary objectives were to similarly assess and compare both groups for taste alteration as well as further functionality problems (FP), psychological and QoL changes. Moreover, the operator’s reporting of any adverse effects or drop out of treatment was taken into consideration.

PROMs were utilised in our study to allow the efficacy of the clinical intervention to be measured from the patients’ perspective as well as the clinical effectiveness and safety measurements [56,57]. All the evaluation questionnaires used to carry out the data analysis have fulfilled the outcome domains of the Initiative on Methods, Measurement, and Pain Assessment in Clinical Trials (IMMPACT) [58].

#### 2.1.1. Patient Cohort

A total of 18 Caucasian subjects (male and female) with various symptoms’ onset times/durations, ranged between 12–188 months (mean 57.50 months) and a total of 10 Caucasian subjects (male and female) with various symptoms’ onset times/durations, ranged between 18–63 months (mean 38.90 months) were recruited for PBM group and MED group, respectively, according to the eligibility criteria. The study cohort had primary neurological BMS (pnBMS) and OINI [Trigeminal nerve (V3); post-traumatic nerve injury of the inferior alveolar nerve or lingual nerve]. This cohort had blood tests prior to the recruitment process to exclude any underlying conditions. These patients were diagnosed in accordance with the International Classification of Headache Disorders, third edition-beta (ICHD Beta 3) diagnostic criteria [9] and invited to participate in the PBM study by letter. The study was conducted and data were collected at the King’s College Hospital NHS Foundation Trust in London, United Kingdom.

#### 2.1.2. Population (P), Intervention (I), Comparison (C), and Outcome (O)—PICO

**P:**  Adult aged ≥ 18-year-old diagnosed with NP, as a result of pnBMS and OINI (V3), according to ICHD Beta 3 [9].
**I:**  λ810 nm laser-PBM or pharmacological treatment modality.
**C:**  Pharmacotherapy versus laser-PBM.
**O:**  Patient-self-reported pain, physical and psychological functionality, QoL, any reported adverse effects and treatment compliance.

#### 2.1.3. Overall Inclusion and Exclusion Criteria for Both Groups (PBM and MED)

##### Inclusion Criteria

1. Subjects of both genders aged ≥ 18 years old with ongoing NP diagnosed according to the ICHD Beta 3 [9].
2. Subjects who were diagnosed with oral iatrogenic neuropathy (OIN) (inferior alveolar nerve or lingual or mental nerve after any dental interventions (mandibular third molar surgery or dental implant intervention).
3. Subjects who were diagnosed with pnBMS according to the ICHD Beta 3 BMS (idiopathic without clinical or laboratory test abnormalities) [9].
4. Subjects with symptom duration ≥ 3 months with normal appearance of intraoral mucosa.
5. Subjects with no physiological or systematic conditions, contributing to the pain.
6. Subjects who had never had phototherapy prior to study enrolment.
7. Subjects volunteering to enrol to either: MED or PBM group based on their wish.
8. Subjects willing to participate in the study from the recruitment process to the end of the protocol.
9. Subjects with controlled systematic diseases with American Society of Anaesthesiologists (ASA) Classification I, II.

##### Exclusion Criteria

1. Elicited pain or without pain
2. Subjects with symptoms’ duration < 3 months.
3. Subjects who have BMS due to other underlining conditions [secondary BMS: BMS by local factors (lfBMS), BMS by systemic factors (sfBMS)].
4. Subjects on medications and whose symptoms were improving.
5. Pregnant and lactating women.
6. Subjects with intraoral mucosal lesions/conditions.
7. Subject with the following neuropathic orofacial pain: Trigeminal neuralgia, glossopharyngeal neuralgia, temporomandibular joint dysfunction syndrome, migraine, odonatological and head and neck origin.
8. Subjects with systematic diseases/or on medications induce NP.
9. Subjects with neurological disorders or autoimmune disorders.
10. Subjects with parafunctional habits and intra-oral trauma.
11. Subjects with hyposalivation related to Sjogren syndrome (unstimulated saliva production ≤ 0.1 millilitres/minute) or any predisposing factors not related to BMS.
12. Subjects who were smokers or had stopped smoking < 6 months prior to enrolling in the study.

#### 2.1.4. Specific Inclusion Criteria for PBM Group

The study included subjects who have had medications with no symptom’ improvement, and who stopped the medications at least 3 months prior to enrolling in the study.

#### 2.1.5. Treatment Protocols

**Figure 2:**
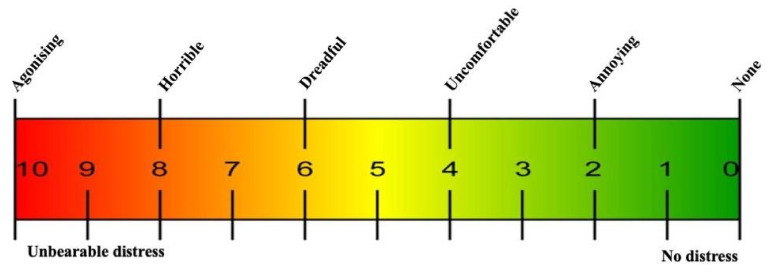
Shows the visual analogue scale (VAS) utilised throughout the treatment and follow-up time points [59].

In the PBM group, the visual analogue scale (VAS) pain score [59] (Figure 2) was assessed and documented pre-treatment (T0), mid-treatment (T1) and end-treatment (T2) for all subjects by a self-report form. Then, a follow-up telephone call one-month (T3), three-months (T4), six-months (T5) and nine-months (T6) after treatment completion was used for the VAS pain score evaluation. For the MED group allocated patients, the VAS pain score was assessed at the clinic at T0, T3, T4, T5 and T6.

The FP questionnaire and scoring scale [60] (Table 1) was assessed at T0, T1 and T2 for all subjects by a self-report form. Then, follow-up telephone calls were conducted at T3, T4, T5 and T6 after completing the treatment evaluating the FP improvement in the PBM group, whereas T0, T3, T4, T5 and T6 were based on subjects’ self-reported at the clinic in the MED group.

**Figure 3:**
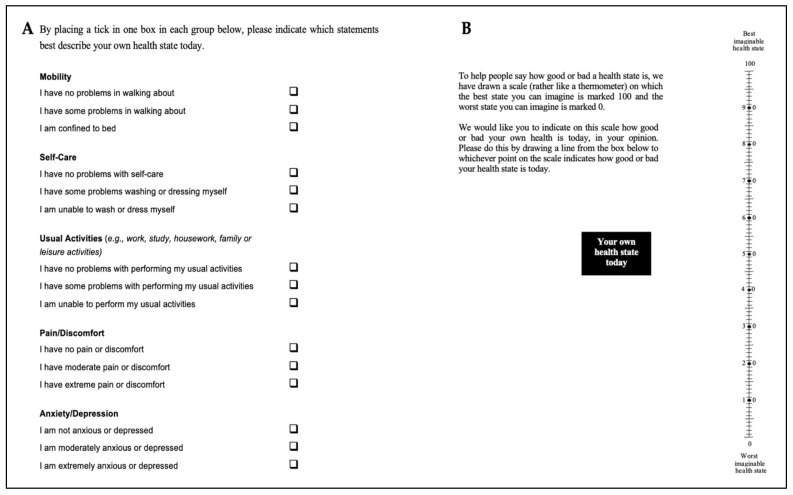
Shows the European Quality of life (EuroQol) Group, 5 dimension 5 levels (EQ 5D-5L) questionnaires. Based on combining supplementary measures that all capture aspects related to the QoL. The EQ-5D-5L questionnaires are based on qualitative and quantitative measures. (**A**) The variables’ questions and associated levels. (**B**) Overall general health percentage [61].

In terms of a psychological assessment tool and overall QoL, European QoL Group (EuroQol) 5 dimensions 5 levels questionnaire (EQ-5D-5L) [61] (Figure 3) was utilised, as it is a valid and reliable instrumental tool and was administered to all the recruited subjects at T0, T1 and T2 by a self-report form. Then, follow-up telephone calls at T3, T4, T5 and T6 were conducted after completing the treatment evaluating the QoL measures, whereas in the MED group the T0, T3, T4, T5 and T6 were based on patients’ self-reported at the clinic.

##### PBM Group Protocol

**Figure 4:**
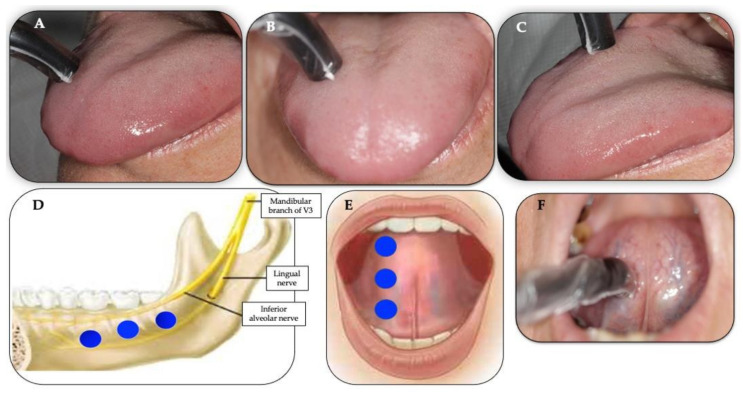
(**A**–**F**) Illustrates the position of the 810 nm laser intraoral single prob applied at 90° (perpendicular) with <1 mm distance from the target tissue as well the allocation and number irradiated points on the trigger and affected points, using “spot technique”. The blue circles illustrate the number and allocation of the points irradiating the affected areas. Clinical photos (**A**–**C**) shows the following 3 points on the anterior two-thirds of dorsal tongue: anterior (**A**), middle (**B**) and posterior (**C**) of right side of the dorsal tongue; clinical photo (**D**) shows the distribution of 3 points on the affected areas along the distribution of the inferior alveolar nerve and lingual nerve along chorda tympani nerve (sensory branch of the facial nerve); photos (**E**,**F**) illustrate the application of the PBM irradiation on the affected areas on the ventral surface of the tongue where the lingual and chorda tympani nerves are distributed). Clinical photo (**F**) shows the application of single laser probe at 90° to the ventral surface of the tongue, irradiating the middle point, whereas assembled photo (**E**) shows the allocation of the 3 irradiated points: anterior, middle and posterior of right ventral surface of the tongue.

A total of nine intra-oral trigger and affected points were irradiated (Figure 4A–F) with λ810 nm at a therapeutic power output of 200 mW [measured by the PM160T-HP power meter (ThorLabs, Bergkirchen, Germany)] for 30 s per each point in a continuous emission mode (CW) at a distance < 1 mm from the target tissue (non-contact), twice a week for five consecutive weeks. The total number of treatments was ten. Table 2 illustrates the PBM protocol and laser parameters calculations. 

A single probe was applied to the target tissue at 90° (Figure 4A–F). The total number of the trigger and affected points was nine and the distribution of these irradiated points was as follows: three trigger points on the anterior two-thirds of the dorsum of the tongue (left or right, depending on each patient’s symptoms) (Figure 4A–C), three irradiated points on the affected areas of the lingual gingivae of the lower posterior teeth along the anatomical distributions of the V3, lingual nerve and chorda tympani (Figure 4D) and three irradiated points on the affected area of the ventral surface of the tongue along the distribution of the lingual nerve and the chorda tympani (Figure 4E,F). It is noteworthy that the chorda tympani nerve is responsible for taste, which carries pre-synaptic parasympathetic fibres joining the lingual nerve (branch of V3).

This protocol was based on the analysis of the best available evidence-based practice in the literature [35,41,42,43,44,45,46,47,48,49,50,51]. A single operator, who is a senior specialist clinician with a wealth of experience in PBM, performed the laser treatment. The questionnaires of the assessment tools listed in subheading 2.1.5 were administered in interview form at baseline (T0) mid-treatment (T1) and at the end (T2) of each treatment session and during the follow-up time points (T3 at one-month, T4 at three-months, T5 at six-months and T6 at nine-month after the PBM intervention period). The data were recorded and stored by a senior laser nurse on Microsoft Excel.

##### MED Group Protocol

The current National Institute for Health and Care Excellence (NICE) guidelines for pharmacological management of NP [24,62] suggest the utilisation of Amitriptyline, Duloxetine, Gabapentin or Pregabalin, as an initial line of NP treatment with switching if the first, second or third drug tried was ineffective or un-tolerated. Hence, the effective dose should be individualised, according to patient response and tolerability. Table 3 illustrates the implemented treatment protocol. The same measuring tools utilised in the PBM group were employed in the MED group to evaluate the variables at baseline and for the follow-up protocol. However, for the MED group, the T1 and T2 time points were unrecorded due to an expected high variance in initial pharmacokinetics based on the large heterogeneity in medications prescribed to the recruited subjects. 

The initial benefits of some of the above-mentioned medications can be noticed within the first or second week of the treatment course; however, it may take up to two months for full effects (last accessed 29 January 2022, www.nhs.uk, www.britishpainsociety) [63,64,65]. Based on this and the hospital review appointment system, the subjects were followed up at time points T3, T4, T5 and T6 (at the clinic), which were comparable with the PMB group. The data were recorded and stored by a senior laser nurse on Microsoft Excel. A single experienced clinician treated the recruited subjects and performed follow-up with them throughout the study period. 

### 2.2. Measures and Tools Used to Evaluate Outcome Variables

#### 2.2.1. Pain Intensity Assessment 

A traditional paper format VAS was used to evaluate pain intensity [59] as it has been shown to be an accurate, valid, reliable and reproducible instrument for subjective rating for pain intensity [66,67]. It is a psychometric scale based on 10 cm lines anchored at the ends by words that define the bounds of various pain dimensions (Figure 2). The patient was asked to place a vertical mark on the scale, indicating the pain intensity level which was assessed verbally as follows: none-annoying-uncomfortable-dreadful-horrible-agonising, or numerically on a 0–10 scale as shown in Figure 2.

#### 2.2.2. Functionality Problems Questionnaire and Scoring

The questionnaires’ scale utilised in this study was adopted from the functionality pain scale (FPS) [60] and modified to address the functional disabilities that subjects might experience, specifically due to NP. As the FPS incorporates both subjective and objective components to assess pain, based on the pain’s perceived tolerability and interference with functioning, this study adapted scale, should therefore be equally valid and reliable in achieving the required data (Table 1). The FP scoring scale used range from zero to ten (0 = no interference; 10 = complete interference).

#### 2.2.3. Psychological Assessment Tool and Overall QoL

The European QoL Group (EuroQol) 5 dimensions 5 levels questionnaire (EQ-5D-5L) provides a generic QoL measure based on combining supplementary measures that capture all aspects related to QoL [61]. The EQ-5D-5L questionnaires are based on qualitative and quantitative measures (Figure 3A).

EQ 5D-5L includes a VAS (EQ-VAS) allowing the subjects to score their general health on a thermometer style scale rated from 0 (worse imaginable health) to 100 (best imaginable health). It describes the levels of each dimension (Figure 3B). The labels for the five levels (5L) are “no problems”, “slight problems”, “moderate problems”, “severe problems” and “extreme problems” for all five dimensions (5D); mobility, self-care, usual activities, pain/discomfort, and anxiety/depression. 

### 2.3. Data Analyses and Statistics 

Data for both PBM and MED groups was collected blinded as described above and unblinded for statistical analysis by an independent collaborator. Data analysis and suitable statistical tests were performed using GraphPad Prism version 9.0.1 for Windows, GraphPad Software, San Diego, CA, USA, www.graphpad.com. The statistical tests applied to any set of data very much depended on the type and distribution characteristics of that specific dataset and are consistently stated within the result section. An overall unbiased two-sided approach was chosen for all statistical tests.

## 3. Results

### 3.1. Trial Study Populations, Demographics and Baseline Characteristics

**Figure 5:**
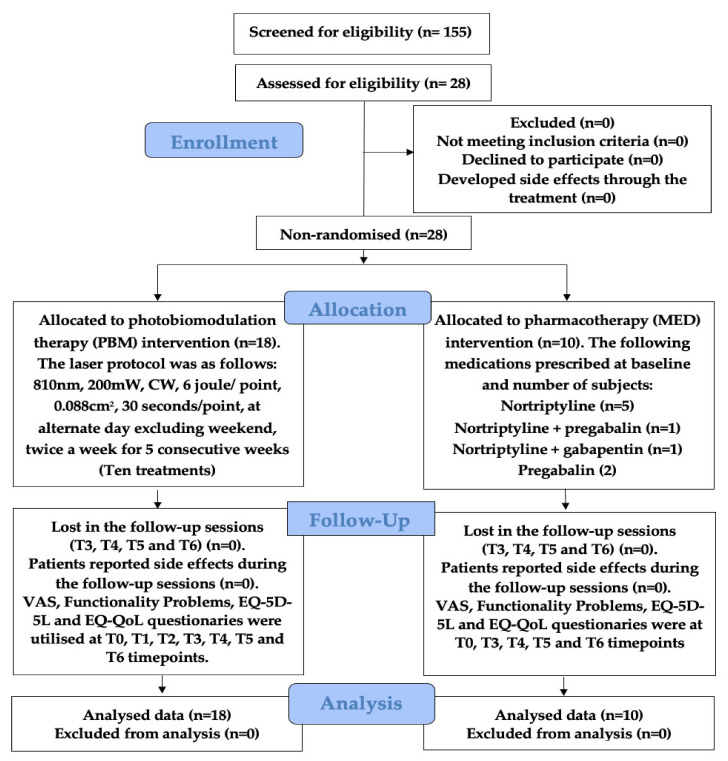
Illustrates the CONSORT diagram flow of subjects’ enrolment, allocation, follow-up and analysis for both groups: PBM and MED, including the treatments and follow-up protocols. Abbreviations: T0: baseline (Pre-treatment); T1: mid-treatment; T2: end-treatment; T3: one-month; T4: three-months; T5: six-months; T6: nine-months; VAS: visual analogue scale; EQ-5D-5L: European-QoL-5 dimensions 5 levels; mW: milliwatt; CW: continuous emission mode; n: number (Supplementary File S2).

Upon ethics approval by New Clinical Procedures Committee (NCPC) at King’s College Hospital NHS Foundation had been granted, the study took place between June 2016 and January 2019 in agreement with NCPC and was registered 177 NCPC. The interventional procedure number IP1547 was obtained from the National Institute for Health and Care Excellence (NICE), England. Out of the 155 screened informed chronic orofacial NP-patients, diagnosed with either BMS or OIN and recruited for trial participation, 28 patients both consented and met the eligibility criteria, all of which completed the study. 18 study participants were allocated to the PBM group and 10 to the paralleled analgesic pharmacotherapy MED group. Figure 5 illustrates a detailed breakdown of patients enrolled in the trial flow. None of the patients reported any adverse effects throughout the 10 treatments.

**Figure 6:**
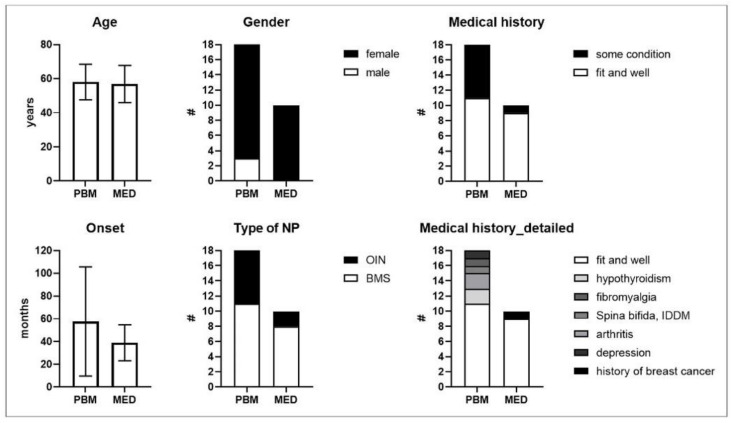
Shows types of self-reported previous or ongoing and medical conditions representing a confounder risk in both test (PBM) and comparison (MED) groups. Abbreviations: PBM: photobiomodulation; MED: pharmacotherapy; #: number of patients; IDDM: insulin-dependent diabetic mellites.

Although no randomisation method was used to assign patients to both study groups, baseline demographics and baseline characteristics of those study populations was not found to be significantly different (Table 4). Contingency analysis could thus not reveal any disproportional allocation over respective PBM- and paralleled MED-intervention groups in terms of; gender (83.33% and 100% female), NP type (61.11% and 88.00% BMS), or the existence of any underlying controlled disease condition (38.89% and 10.00%). These conditions were as follows: a history of breast cancer, hypothyroidism, arthritis, fibromyalgia, spina bifida and depression (Figure 6). The overall representation of BMS- and OIN-diagnosed patients for the complete study cohort was 67.86% and 32.14%, respectively. The patients allocated to the PBM study group also compared in age (mean ± SD = 58.00 ± 10.39 years) to those patients assigned to the MED group (mean ± SD = 56.80 ± 10.84 years). Although there was a vast spread in the duration that patients had already suffered from chronic NP, the number of months passed since onset until trial intervention was also not statistically different between study groups (PBM group mean ± SD = 57.50 ± 47.93 months versus MED group 38.90 ± 15.77 months). The above study population demographic and baseline characteristic comparisons therefore justify further explorative or qualitative outcome comparisons between paralleled study groups.

### 3.2. Patients’ Acceptance of Treatments

There were no adverse events observed or reported in both groups during the treatments and throughout the follow-up time points and there were no dropouts from the study (Figure 5). For the MED group, however, Table 3 shows that the doses of analgesic medications needed to be increased for all 10 patients at the three-month (T4) follow-up evaluation and 2/10 patients were even prescribed additional medication for pain management. This indicates that for those patients recruited to the MED group, the initial doses of the medications had become ineffective to reduce the pain intensity below a tolerable level between 1–3 months after prescription. In particular, the prescribed mediation dose of Nortriptyline increased from 10 mg at T0 to 20 mg at T4 (3/10 subjects) or from 20 mg to 40 mg (2/10), whereas for one subject who was initially on 10 mg Nortriptyline and 100 mg Pregabalin, the doses increased to 20 mg and 200 mg, respectively. In addition, the initial doses of 100 mg and 25 mg Pregabalin for two subjects also needed to be doubled at T4. The two subjects, who were on 600 mg Gabapentin, were additionally prescribed Nortriptyline at the T4 time point. 

All the above-mentioned medication regimens remained unchanged thereafter at the six-month (T5) and nine-month (T6) follow-up time points. In contrast, none of the 18 PBM group subjects was in need of additional analgesic medication throughout the five-week intervention period and nine-month follow-up period, indicating a long-lasting, neuro-regenerative, beneficial effect on oral NP by the PBM procedure.

### 3.3. Self-Reported Maximum Pain Intensity Score (PIS_max_)

**Figure 7:**
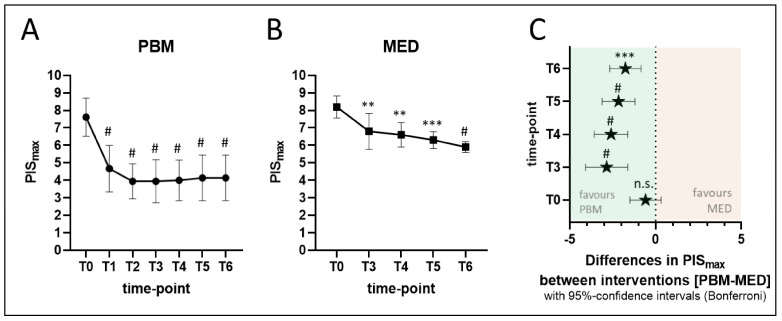
(**A**–**C**) Shows self-reported highest pain intensity score (PIS_max_). (**A**) shows PIS_max_ (mean ± SD) over time in the PBM group (circles) and (**B**) shows PIS_max_ (mean ± SD) over time for the parallel MED group (squares) on a visual analogue scale from zero to ten cm. (**C**) shows mean differences in PIS_max_ between both interventions and the 95%-confidence intervals as the result of a full mixed-effects two-way ANOVA analysis, demonstrating analgesic efficacy in favour of the PBM-treatment throughout the study period. Bonferroni’s multiple comparison statistics: *p*-value indications: n.s. = not significant; ** < 0.01; *** < 0.001; # < 0.0001. In (**A**,**B**) (means ± SD): *p*-value indications show the significance level of pairwise differences between means of the respective time points with T0. Abbreviations: PIS_max_: self-reported highest pain intensity score; T0: baseline; T1: mid-treatment; T2: end-treatment; T3: one-month; T4: 3 months; T5: 6 months; T6: 9 months.

PBM significantly alleviated self-reported highest pain intensity (PIS_max_) on a visual analogue scale from zero to ten cm, already with −2.94 cm (*p*-value < 0.0001) at mid-treatment (T1) and with −3.67 cm (*p*-value < 0.0001) at end-treatment (T2), as compared to baseline (T0: PIS_max_ = 7.61 ± 1.09 cm). Interestingly, this fast reduction in pain sensation remained stable and highly statistically significant throughout the follow-up assessment period (*p*-values at T3 to T6 below 0.0001) with the therapeutic benefit still being as high as a −3.47 cm reduction on the VAS at the nine-month follow-up assessment time point (T6: PIS_max_ = 4.14 ± 1.30 cm) (Figure 7A). 

Moreover, for the parallel MED group, a less pronounced but significant—seemingly linear—reduction on mean PIS_max_ was also observed for consecutive intervention follow-up time points T3: −1.40 cm (*p*-value = 0.0014), T4: −1.60 cm (*p*-value = 0.0020), T5: −1.90 cm (*p*-value = 0.0002) and T6: −2.30 cm (*p*-value < 0.0001) as compared to the baseline assessment at (T0: PIS_max_ = 8.20 ± 0.63 cm) (Figure 7B). 

This indicates that PBM and pharmacotherapy analgesic effect dynamics are presumably substantially different with a faster, more pronounced and long-lasting benefit in favour of the medication-free PBM intervention. Even though analgesic medication doses were raised at T4 for all subjects in the MED group (Table 3), the gradual reduction in PIS_max_ by this intervention could not parallel the improvements reported by the PBM group at any time point assessed. Indeed, when comparing PIS_max_ in the follow-up period between the paralleled intervention groups by a full mixed-effects two-way ANOVA analysis, PBM was initially −2.86 cm (*p*-value < 0.0001) more effective than standard of care medication therapy at T3, despite the fact that the baseline mean PIS_max_ values at T0 were not different between both groups (*p*-value = 0.4124). Figure 7C furthermore shows that mean differences in PIS_max_ between both interventions remained in favour of the PBM treatment throughout the subsequent follow-up period with the therapeutic benefit still remaining a significant −1.76 cm (*p*-value = 0.0001) at T6, the nine-months follow-up assessment time point. 

### 3.4. EQ-5D-5L Indices

**Figure 8:**
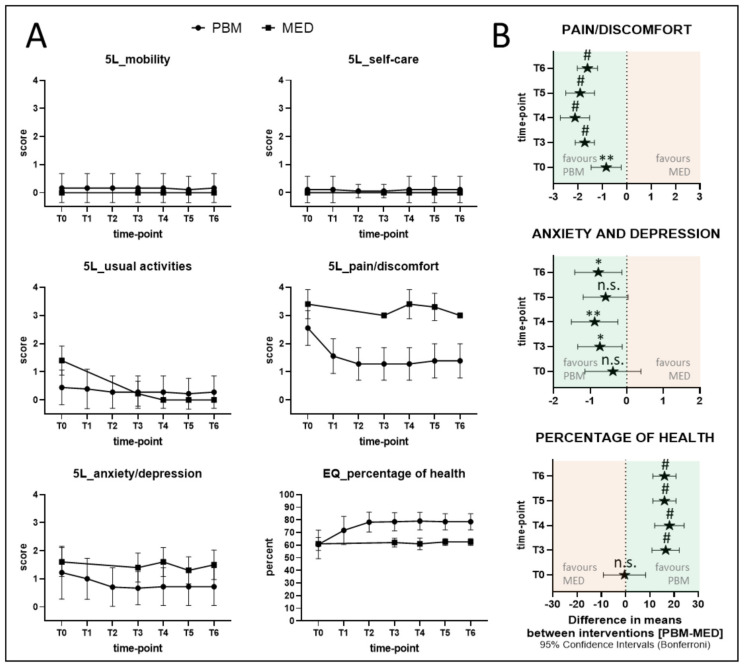
(**A**,**B**) Shows the results of EQ-5D-5L indices for PBM and MED groups for the different assessment time points. (**A**) Shows the self-reported EQ-5D-5L indices as mean (± SD) and aligned for paralleled intervention groups PBM (circles) and MED (squares). (**B**) Depicts mean differences between the interventions (±95%-confidence intervals) at different time points of selected EQ-5D-5L indices, for which a main-effects analysis revealed a significant group and time dependency, along with *p*-value indications for Bonferroni’s multiple comparison statistics of the full mixed-effects model: n.s.: not significant; * < 0.05; ** < 0.01; # < 0.0001. Abbreviations: T0: baseline; T1: mid-treatment; T2: end-treatment; T3: one-month; T4: three-months; T5: six months; T6: nine-months.

The EQ-5D-5L Quality-of-Life (QoL) assessment was introduced by the EuroQol Group in 2009 (last accessed 29 January 2022, http://www.euroqol.org) and essentially consists of both the EQ-5D descriptive system and the EQ-VAS [68,69,70]. The descriptive system comprises five dimensions: mobility, self-care, usual activities, pain/discomfort and anxiety/depression. Each dimension has five levels: no problems (0), slight problems (1), moderate problems (2), severe problems (3) and extreme problems (4). The EQ-VAS records the patients’ self-rated health on a vertical VAS, where the endpoints are labelled ‘The best health you can imagine’ (=100%) and ‘The worst health you can imagine’ (0%). Figure 8A,B describes the detailed results of both groups at different time points.

### 3.5. Extended QoL-Assessment-Functional Indices

Besides the general EQ-5D-5L, a more in-depth QoL assessment was performed by patients’ self-grading their overall pain interference with oral health-related functional indices on a scale from zero (=no interference) to ten (=complete interference) (Table 1) [60]. These functional indices were speech, pronunciation, confidence, eating, drinking, brushing teeth, taste, smell, kissing, make-up application/shaving, social events, family relation, sleeping and work. Additionally, the total FP score was calculated as the mean pain interference grade of all functional parameters for each given patient and time point. 

**Figure 9:**
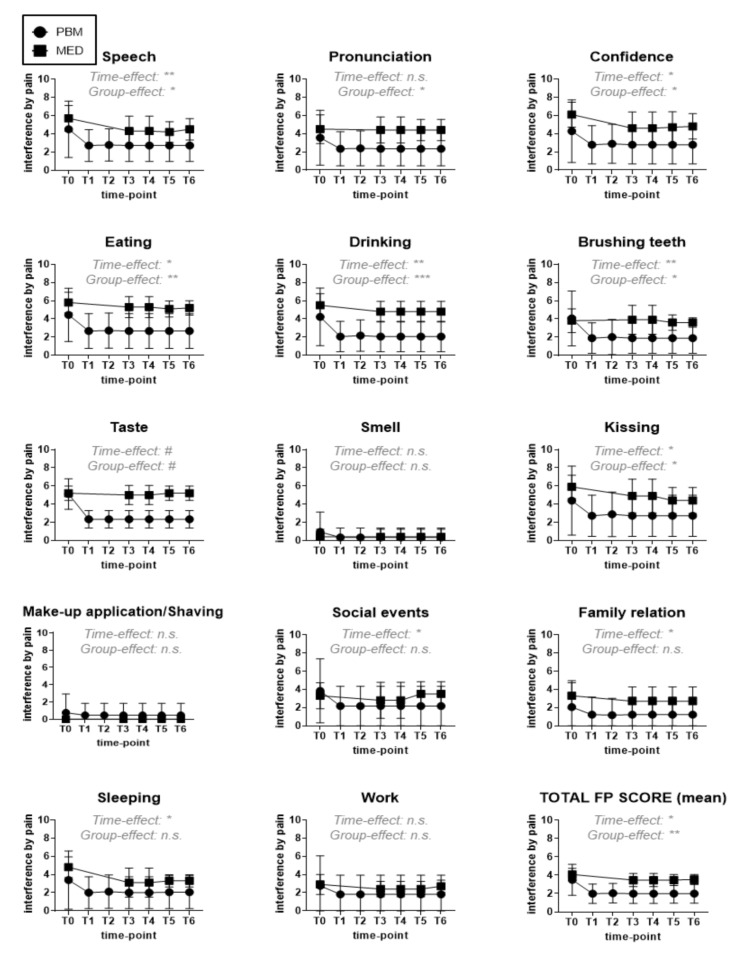
Pain interference with 14 functional indices as experienced and self-reported by the study participants on a scale from zero (=no interference) to ten (total interference or complete loss of functionality), allocated to either PBM (circles) or MED (squares) intervention groups. The functional parameters assessed, relating to QoL, are depicted above in each graph, as well as the significance level of the main effects by ANOVA analysis (Group-effect = intervention dependency, Time-effect = time-point dependency) for the respective functional parameter in the study (n.s. = not significant; * < 0.05; ** < 0.01; *** < 0.001; # < 0.0001). Abbreviations: T0: baseline; T1: mid-treatment; T2: end-treatment; T3: one-month; T4: three-months; T5: six-months; T6: nine-months.

Pain caused by BMS or OIN interfered considerably with the functional parameters assessed, except for *work*, *make-up application/shaving* and *smell*, thereby validating the relevance of this in-house QoL assessment for oral NP patient studies (Figure 9). Statistical main effects analysis revealed that functionality-based QoL of these patients could be improved by the paralleled therapeutic interventions in this study (*p*-value = 0.0151), with PBM-treated patients being significantly more responsive (*p*-value = 0.0022), based on the lower corresponding total FP scores. In particular, observed improvements (i.e., of those pain-related functional indices with a time-point dependent significant effect), *speech*, *confidence*, *brushing teeth*, *kissing*, but most obviously *eating* (*p*-value = 0.0018), *drinking* (*p*-value = 0.0006) and *taste* (*p*-value < 0.0001) were primarily achieved with PBM, whereas the type of intervention did not significantly matter for *social events*, *family relation* or *sleeping* (*p*-value ≥ 0.05).

## 4. Discussion

NP can be challenging to treat effectively [1,71]. Pharmacotherapy can reduce pain, but the majority of patients do not experience complete pain relief [71,72]. Hence, it is highly debilitating, profoundly distressing and may negatively affect the patient’s QoL [73,74]. 

Our prospective parallel study has demonstrated the efficacy of λ810 nm PBM in modulating NP intensity and improving functionality and QoL at mid- and end-treatment sequence and sustained throughout the follow-up time points at one, three, six and nine months, comparing to or exceeding the gold standard-of-care pharmacotherapy, while being safe and well accepted by the patient. This clearly shows the most important finding of our study. To the authors’ best knowledge, this is the first comprehensive parallel study (PBM and pharmacotherapy) in treating NP, induced by BMS and OIN, of a Caucasian cohort based on a long-term follow-up period of nine months.

The results of published clinical studies to date are inconsistent in NP management. Despite the positive outcomes of the current published RCTs [35,41,42,43,44,45,46,47,48,49,50,51], utilising PBM in alleviating NP intensity in patients with pnBMS, a lack of high methodological quality and a standardised laser-PBM protocol is repeatedly noted in recent systematic reviews and meta-analyses [52,53,54]. The questions raised by previous studies and reviews have served us to structure our current study laser-PBM protocol and to employ a robust methodology, in order to contribute to achieving a consensus in PBM therapy protocol for pnBMS management, which is essential for replication in future studies. We have outlined the key findings of our study to support the abovementioned statements.

### 4.1. Demographic Characteristic of Study Cohort

It is interesting to note that the majority of the subjects who enrolled in our study were female and middle-aged as was supported by the literature [35,75,76].

In our cohort, the affected area was within the anterior two-thirds of the tongue, either right or left lateral dorsal tongue. This was also in agreement with the literature, reporting trigeminal small-fibre sensory neuropathy in pnBMS was evidenced by diffuse degeneration of epithelial and sub-papillary nerve fibres of tongue anterior two-thirds [7] and lateral borders of the tongue to be the sites most commonly affected by pnBMS [77,78].

### 4.2. Evaluation of the Study’s Laser-PBM Protocol and Its Parameters

There is a wide range of PM doses (fluences) utilised in the available published RCTs [35,41,42,43,44,45,46,47,48,49,50,51], ranging from 1–200 J/cm^2^, whereas the range of power outputs irradiated by different wavelengths ranged from 30 mW–1 W and λ660–λ980 nm, respectively, where CW was utilised in ten of the 12 studies [35,42,43,44,45,46,48,49,50,51]. The exposure time, frequency and average total number of treatment sessions have ranged from 4–381 s/point, 1–5 sessions/week and 10 sessions, respectively.

It is noteworthy that only three out of 12 studies utilised a power meter to measure the therapeutic power output [35,46,49]. All studies showed positive results except for a study conducted by Pezelj-Ribarić et al., 2013 [35]. Moreover, only one study [44] compared PBM to clonazepam (mouthwash), whereas the remaining 11 studies compared PBM to placebo. The number of sessions per week, time interval and duration of treatment varied among the studies, as follows; daily for ten days (10 sessions), once per week for 4 weeks (4 sessions), twice a week for 4 weeks (8 sessions), once a week for 10 weeks (10 sessions), twice a week for 5 weeks (10 sessions), six times a week for 4 weeks (24 sessions).

Taking the aforementioned observations into consideration, we formulated a standardised therapeutic PBM protocol, proven effective in this study (therapeutic wavelength of λ810 nm and at a power meter verified output of 200 mW, spot size 0.088 cm^2^, 6 J/point, 9 points, 30 s/point, 68.1 J/cm^2^, twice a week for five consecutive weeks, ten sessions in total). Additionally, despite three studies [35,46,49] that utilised a power meter to measure their therapeutic power outputs, their findings showed variation in their clinical outcomes. Their therapeutic power outputs and utilised wavelengths were as follows: 100 mW, 830 nm [46] and 200 mW, λ808 nm ± 5 nm [49] and showed positive findings in pain intensity reduction but marginal improvement in psychological status, whereas a study conducted by Pezelj-Ribarić et al., 2013, utilised a therapeutic power output of 30 mW, λ685 nm but did not obtain significant results [35]. 

High fluences (doses) can offer more beneficial effects for pain relief due to the multiphasic PBM dose response compared to generally accepted biphasic dose response [79,80,81]. Moreover, wavelength plays a key role in regulating the penetration depth of the laser irradiance in the tissue [82,83], noting that the λ808 nm light penetrates as much as 54% deeper than λ980 nm [84]. Therefore, 810 nm could be a suitable wavelength in the management of NP. It is important to take into consideration the treatment frequency and time interval as well [13].

### 4.3. Representation of the Study’ Outcome Measures and Their Influences to Determine PBM Efficacy

In order to achieve effectiveness in NP management by capturing all of the outcome domains, valid and reliable standardised outcome measurement tools are required. 

Previous RCTs studies have used various methods of assessment to evaluate the primary and secondary outcomes. All of these studies utilised VAS to evaluate PIS and Oral Health Impact Profile-14 (OHIP-14) [42,44,46,50] and its various variations employed in other studies such as: Italian [41], Persian [43] and Croatian [48] to assess pain, physical and emotional impairment, whereas one study used the hospital anxiety–depression scale (HANDS) to assess anxiety/depression [49]. The remaining four studies [35,45,47,51] have not addressed this outcome domain, however, three of them assessed the salivary flow (functional problem) of which two [35,47] used immunohistochemical analysis, whereas one study conducted by Scardina et al., 2020 used video capillaroscopy [51].

In our study, we have evaluated all the NP outcome domains using the following assessment tools: VAS to assess pain intensity, 14-indices FP questionnaires to assess pain-interference with daily functions and EQ-5D-5L, to assess pain, psychological status, including health-related behaviours (anxiety/depression) and health-related QoL. PROMs prevent any possible bias in the evaluation by the trials examiners and are valid and reliable measurement tools in idiopathic NP [56,57,85] (https://www.gov.uk/government/publications/patient-reported-outcome-measures-proms-in-england-a-methodology-for-identifying-potential-outliers-2, last accessed 29 January 2022). We have followed the recommendations of IMMPACT-II, considering the evaluation of six domains to measure the outcomes in clinical trials involving the management of chronic pain [58,86] therefore the results of our study were robust and reliable.

### 4.4. Representation of the Treatment Outcomes for Both PBM and MED Groups

#### 4.4.1. Evaluation of the Maximum Pain Intensity Score (PIS_max_) Reduction

An RCT study conducted by Arduino et al., 2016 [44] evaluated the effect of λ980 nm PBM (300 mW, 10 J/cm^2^, 10 s/point, 2 mm light-tissue distance, twice a week for five consecutive weeks) versus clonazepam in patients with BMS. The results showed that PBM was superior to clonazepam in improving PIS on VAS and was statistically significant over the three-month follow-up period without a significant improvement in anxiety/depression. A recent RCT conducted by de Pedro et al., 2020 [50] utilised λ810 nm (0.6 W, 6 J/point, 10 s/point, total 56 points, 0.5 cm^2^, 2 mm light-tissue distance, twice a week for five consecutive weeks) to evaluate the effects of PBM versus placebo. It concluded that PIS reduction at the end of treatment remained the same among 90% of study subjects (*n* = 9) in the PBM group at the four-month follow-up period and significant psychological improvement was noted at end-treatment and maintained at the one- and four-months follow-up period in the PBM group. It is noteworthy that this implies that in our present study, PBM already almost fully modulated pain intensity by the 5th session [mid-treatment, (T1)]. The authors, however, assume that based on previous observational practice, later PBM sessions (5th to 10th) might still be useful to stabilise the effects of treatment outcomes. Our study was the first to validate the efficacy of PBM on NP for a longer follow-up period of up to nine months. In contrast to the other studies where the follow-up period varied from 4–16 weeks [35,41,42,43,44,45,46,47,48,49,50,51], our results demonstrate sustained beneficial effects by PBM for even up to nine months post-treatment, indicating a pro-regenerative effect of PBM on the TN pathways in NP. 

In our study, despite the severe and persistent nature of the symptoms at baseline with onsets predating an impressive 57.50 ± 47.93 months in the PBM group, a notably fast reduction in PIS_max_ on VAS from 7.6 (=dreadful to horrible) at baseline (T0) to 3.9 (=uncomfortable) at one-month post-treatment (T3) could be achieved. On the other hand, mean PIS_max_ was only reduced from 8.2 (=horrible) at baseline to 6.8 (=dreadful) at T3 in the MED group. At this point, it is worth mentioning that the subjects of the PBM group have had pharmacotherapy previously without benefits and stopped at least three months prior to enrolling in the study.

The pharmacotherapy group in our study showed a slower and gradual but significant reduction in PIS_max_ on VAS in response to medications with single or combined drugs that needed to be up-dosed at the T4 time point. Although here we do not present the data to support it, it is possible that the pharmacotherapy provides a rather late attenuation of NP. This is in agreement with the results of an RCT parallel-placebo study by Serpell et al., 2017 [87], demonstrating that many patients do not respond to pregabalin at lower doses, however, subsequently responded when the dose was increased to achieve an optimal analgesia. Another study showed that pregabalin at doses ≥ 300 mg/day was more effective in improving pain than pregabalin at doses ≤ 150 mg/day [88]. This was confirmed by a recent systematic review published in the Cochrane Database in 2019 [89]. However, the higher doses also significantly increased a number of adverse events for which the treatment had to be discontinued [64,89,90]. In contrast to this, we assume a more robust, pro-regenerative and lasting effect of PBM at energy doses that are far below the threshold for damage and therefore virtually free of side effects or adverse events.

It is important to mention a non-anticipated confounding factor for three of 18 patients in the PBM group. After these patients showed an improvement in the pain intensity at T1 and T2, a slight elevation occurred at T3. Nevertheless, pain improvement remained superior to the score at T0. These patients self-reported during the T3 assessment time point that the pain intensity increased after they had sustained a severe chest infection and gastric problems (between T2 and T3), presumably unrelated to the interventional treatment of this study but for which Ciprofloxacin had been prescribed for 7–14 days. The authors assume that the reported increase in NP could have been triggered by the remote infection or more likely by the antibiotics used by these patients [91,92]. This would also be in agreement with the fact that NP can be initiated after one or more adverse life events, related to either clinical or social challenges [93]. Additionally, evidence-based practice demonstrated that Fluoroquinolone (Levofloxacin and Ciprofloxacin), but not Amoxicillin, appears to increase peripheral neuropathy risk by 47% [94]. We suggest that further research should be conducted to investigate the causal relation between antibiotics use and NP and its related mode of action.

#### 4.4.2. Evaluation of the Functional Problems’ Improvement

It is noteworthy that, to the best knowledge of the authors, the present study was the first to utilise the detailed 14-indices functionality questionnaires, evaluating the effects of PBM therapy and pharmacotherapy on improving daily functionality related to QoL. There is evidence to suggest that NP can have a great impact on daily functions [95,96,97].

PROMs for functional parameters in our study revealed that both intervention strategies, PBM and pharmacotherapy, could ameliorate pain interference with daily functions such as: *sleeping*, *family relations* and *social events,* but not with *work*. On the other hand, *smelling*, *make-up application* or *shaving* did not seem to be significantly impaired by NP, as was reported by the patients in our study cohort. Interestingly, a statistically significant better improvement by PBM as compared to pharmacotherapy was reported for the following NP-related functions: *speech* and *pronunciation*, *brushing teeth*, *kissing*, *confidence drinking*, *eating* and most obviously; *taste*.

In particular, our results showed a reduction in patient self-reported *taste* alteration at mid-treatment that sustained to end-treatment and throughout the nine-month follow-up in the PBM group, whereas no changes occurred in the pharmacotherapy MED group. To the authors’ best knowledge, our comparative parallel study is the first in the literature, addressing the impact of PBM on dysgeusia, compared to pharmacotherapy. Additionally, our PBM protocol is in agreement with the suggested PBM protocol proposed by two previous studies for curative taste alteration induced by NP [98,99]. Interestingly, the results of an in vitro study conducted by Oron et al., 2007 [100] showed a significant restoration of taste pathway-related ATP production by PBM-treated human neuronal cells in peripheral nerve dysfunction has also been found. This coincides with the histological results of a clinical study conducted by Suarez et al., 2006 [101] and a case reported by El Mobadder et al., 2019 [102]. In this context, it was proposed that a decrease in the tongue gustatory sensitivities on an electrogustometric test on the dorsal surface of the tongue is related to the degeneration of the chorda tympani nerve that leads to trigeminal neuropathy or glossopharyngeal nerve inhibition [103,104]; whereby irradiating the affected areas as well as the trigger points would capture and restore all the symptoms associated with idiopathic NP, ensuring optimal outcomes. This proposition was supported by a systematic review and meta-analysis conducted by Hanna et al., 2021 [105] and is now thus further consolidated by our findings here. Our results confirm that our approach in mapping the triggered and affected areas to avoid a possibility of overlap irradiation during PBM [106,107], taking into consideration either right or left lateral dorsal tongue where there is the prominent presence of taste buds, depending on presented symptoms and irradiating the lingual nerve where it carries along the chorda tympani, is effective, reliable and reproducible.

It is noteworthy that hyposalivation might also have a significant contribution to taste alteration [108]. Hence, the effects of PBM in improving the salivary flow by reducing the salivary levels of TNF-alpha and IL-6, which are proinflammatory mediators found to be elevated in patients with pnBMS [35], can ultimately have a great impact on restoring taste impairment. PBM mediates the healing and regeneration of these taste bud cells and olfactory receptors and can, therefore, stimulate the regeneration of the local neuronal complex [106,109]. Interestingly, the results of our cohort have shown that smell interference by NP was virtually lacking, despite the known dependency correlation between smell and taste impairment [110]. 

In summary, our study PBM protocol proves to be effective and indicative in improving the oral NP-related functionality of this cohort.

#### 4.4.3. Evaluation of the Psychological Status and QoL Improvement of Both PBM and MED Groups

A systematic review and meta-analysis was conducted by Hanna et al., 2021 [111] recommended EQ-5D-5L as a robust standardised assessment tool for pain and QoL evaluations in patients with NP; hence, our study, for the first time, has introduced EQ-5D-5L for QoL-assessments in NP. Our findings with this form of PROMs showed a statistically significant reduction in *pain/discomfort*, *anxiety/depression* and an improvement in general *health percentage* (QoL), evident as early as T1, peaking at T2 assessment and fundamentally sustained throughout the nine-month follow-up time points favouring the PBM intervention group (Figure 8A,B). It is noteworthy that despite the fact that anxiety and depression are reported in patients with pnBMS, such conditions commonly arise only after pnBMS onset [112]. In this context, our results show that the *anxiety/depression* variable significantly reduced when the NP was alleviated, especially in the PBM group.

Controversially, previous RCTs studies that addressed anxiety and depression by evaluating general health, QoL and psychological indices, showed inconsistency in reporting pBMS-specific symptoms when the following outcome assessment tools were employed: short-form health survey (SfF-36), psychometric symptom checklist (SCL)-90-R and McGill Pain Questionnaire (McGill) [44,50]. OHIP-14 [42,44,46,50], including its various variations employed such as: Italian [41], Persian [43] and Croatian [48], HANDS [42,44,49], geriatric depression scale (GDS) [44] and psychometric symptom checklist (SCL)-90-R [50]. With an emphasis on a study by Arduino et al., 2016 [44], they showed that anxiety and depression levels did not change statistically in both laser-PBM and clonazepam (mouthwash form) groups (p* > 0.05) assessed by GDS, OHIP-14, MPQ and HANDS. 

Remarkably, the functional dimensions: *usual activities*, *selfcare*, and *mobility*, showed no significant differences to the values reported at baseline for either treatment group. In this context, our study has shown that EQ-5D-5L is a well-suited, sensitive tool for addressing general physical and psychological health, but not for functional impairments that specifically relate to NP disease. For future studies, the authors therefore suggest adopting the approach followed here to combine generic and disease-specific PROMs, reflecting on health-related QoL.

#### 4.4.4. Evaluation of Subjects’ Acceptance to Treatments of Both Groups (PBM and MED) and Reported Adverse Effects

In our study, there were no adverse events reported for either PBM or pharmacotherapy during treatments and throughout the study period. Moreover, the CONSORT flow chart demonstrated that all participants completed the treatment and follow-up protocol. Opposed hereto, in an RCT study conducted by Arduino et al., 2016 [44] comparing PBM to 2 mg of topically localised oral disintegrated clonazepam tablet application, 32% of the patients treated with clonazepam reported dizziness, fever, headache and a lack of appetite in the course of the 12-week study period, whereas none of the patients treated with PBM reported adverse effects [44]. There is evidence to support that patients have discontinued long-term opioid therapy (especially oral opioids) due to adverse events or insufficient pain relief; however, weak evidence suggests that patients who are able to continue opioids long-term experience clinically significant pain alleviation and inconclusive QoL and functional improvement [20,113].

For the MED group in our study, the doses of analgesic medications needed to be increased after three months for all patients. All of the adjusted medication regimens remained unchanged thereafter up to the last follow-up time point. In contrast, none of the 18 PBM group subjects was in need of additional analgesic medication throughout the whole study period, indicating a long-lasting beneficial effect on oral NP by the PBM procedure. Three patients, allocated to the PBM group, however, suffered from gastric infections at some point during the study, which was assumed to be unrelated to the interventional treatment but required antibiotic treatment with possible confounding effects on the oral pain sensation levels as discussed above. Several studies evaluated the possible adverse effects of PBM, but no significant results were noted or reported [35,41,42,43,44,45,46,47,48,49,50,51]. Our study results are in agreement with this data. Importantly, all PBM group allocated subjects completed the treatments without interruption with a satisfactory response, which was significantly sustained throughout the long-term follow-up of nine months, as was suggested by de Pedro et al., 2020 in a recent systematic review [114] Additionally, our study has fulfilled the criteria of Farag et al., 2019 [58] to qualify as a high-quality study in addressing various outcome domains related to pain, including physical functionality, emotional status, QoL and adverse events. To the best of our knowledge, this study is the first study investigating the efficacy of PBM, as a monotherapy, compared to the gold standard analgesic pharmacotherapy. It is noteworthy that various validated instrumental PROMs pain evaluations were utilised in our study to prevent subjective bias in records. Our study has also shown that the utilisation of both EQ-5D-5L and VAS assessment tools to evaluate NP intensity have demonstrated to be a robust methodological approach to achieve optimal clinical outcomes, which was reflected in our positive findings and exhibited substantially statistically significant in NP alleviation.

### 4.5. Strengths and Limitations of the Study

Our prospective parallel study, for the first, has shown PBM efficacy in NP alleviation with the longest follow-up period of nine months. In terms of PIS_max_ on VAS, a substantial 4-fold reduction from baseline to T3 was observed in the PBM group compared to a 2-fold reduction in the MED group. Moreover, a statistically significant improvement in physical functionality and QoL at T1, progressing at the T2 time point and then profoundly uninterrupted throughout the nine-month follow-up time points in the PBM group compared to the MED group. Our results also demonstrated that the psychological parameters significantly reduced when NP was alleviated, especially in the PBM group. It is important to note that the assessment methods of outcomes were of high quality, ensuring that NP intensity, physical functionality, psychological status, QoL and adverse events were captured. No adverse effects were reported in both groups throughout the treatment course and follow-up time points.

A public health campaign was carried out in order to recruit subjects from across England for the PBM treatment option in our study, resulting in the number of participants and study group allocations being based on patients’ choices and preferences. In addition, telephone follow-up was conducted where it was practically convenient and financially feasible. MED group subjects received standard care based on their choices and their follow-up at the clinic, relatively related to geographical convenience [115]. Therefore, this study had a non-randomised trial design, unequally balanced in terms of group sizes, yet baseline demographics and characteristics did not differ between both study arms. 

Every patient is different in terms of their responses to antidepressants and anticonvulsants medications which are commonly prescribed in treating chronic NP [116,117]. According to the British Pain Society, the use of the following medications, as a single drug in treating chronic NP, might take up to 1–2 weeks to start working and up to 2 months (8 weeks) to exert its full analgesic effects (last accessed 29 January 2022, www.nhs.uk, www.britishpainsociety) [63,64,65]. In this context, the authors expected a high variance in the initial pharmacokinetics for the MED group, based on the large heterogeneity in medications prescribed to the recruited subjects and assumed the most appropriate time points to evaluate pain improvement should be in the follow-up period (T3, T4, T5 and T6). As a result, data for T1 and T2 time points were unrecorded for the MED group for the scientific reasons described above, which thus impedes some direct and mixed-effect comparative analyses with the PBM group. This classifies this two-armed comparative clinical trial as paralleled rather than fully controlled or referenced, and results should therefore be interpreted as such, i.e., focusing on intra- rather than inter-group differences.

## 5. Conclusions and Future Perspective

Our prospective parallel study, for the first time, has demonstrated the efficacy of laser-PBM in modulating NP intensity, improving functionally and QoL at T1 and peaking at T2 and sustained throughout all of the follow-up time points (T3, T4, T5 and T6). Moreover, our study, for the first time, proves laser-PBM as a safe, potent analgesic, antioxidant and regenerative, compared to or even exceeding the clinical benefits of the gold standard analgesics pharmacological approach; thereby, laser-PBM presents an up-to-date first validated, medication-free, alternative therapeutic option for oral NP treatment. Our positive laser-PBM findings further support more patients’ benefits in improving QoL and functional activities, which are considerably impaired by NP such as: *eating, drinking and tasting*, whereas the analgesic medication regimens did not. Furthermore, observed long-term pain relief and functional benefits indicate that laser-PBM modulates NP pathology in a pro-regenerative manner, presumably via antioxidant mechanisms.

Our effective laser-PBM application protocol and reliable investigational tools are relevant, easily reproducible and therefore suited for well-designed, multi-centred RCTs, utilising large data or any other study with the aim to consolidate or optimise PBM effects on oral NP.

## Highlights

1. Our prospective parallel study, for the first time, has demonstrated the efficacy of photobiomodulation (PBM) in modulating oral neuropathic pain (NP) intensity, improving functionality and quality of life (QoL) at mid- and end-treatment that sustained throughout all the follow-up time points (one, three, six and nine months).
2. Our study, for the first time, proves that PBM; a safe, analgesic, antioxidant and pro-regenerative physical therapy, compares to or even exceeds the clinical benefits of the gold standard analgesic pharmacological approach, thus presenting itself as an up-to-date first validated, medication-free, alternative therapeutic option for the treatment of oral NP.
3. Our results furthermore support additional patient’ benefits of PBM by ameliorating the QoL and functional activities which are considerably impaired because of NP, such as eating, drinking and tasting, whereas analgesic medication regimens did not.
4. Our effective PBM application protocol and reliable investigational tools are relevant, easily reproducible and therefore suited for well-designed, multi-centre randomised clinical trials (RCTs), utilising large data or any other study with the aim to consolidate or optimise PBM effects on oral NP.

## Figures and Tables

**Table 1:** Functional problems of 15-questionnaires reflect on patients’ affected daily functions. The scoring of the scale is based level of interface of the function on ranges from 0 = no interference. The score for each item ranges from 0–5 indicates no interference-tolerable, respectively, whereas from 6–10 indicates intolerable/prevention of a number of activities [60].

Are Your Daily Functions Affected?	
Rated on a Scale from: 0: No Interference 10: Complete Interference	Score
Speech	
Eating	
Drinking	
Kissing	
Sleeping	
Smell	
Confidence	
Work	
Family relationships	
Social events	
Pronunciation	
Taste	
Make-up application	
Shaving	
Brushing teeth	
Others (please state)	

**Table 2:** Illustrates the laser device specifications, PBM-therapeutic protocol and laser parameters’ calculations of the study. Abbreviation: 1/e^2^: light beams do not typically have defined edges and the beam distribution is not usually uniform (to calculate the power density, physicians use the mathematical function 1/e^2^ to define the area); NOHD: nominal ocular hazard distance (the distance at which the power output is safe to view without safety spectacles (i.e., below the MPE); 1/e^2^: the spot size is recommended to be used for dosage calculations; J: Joule; s: seconds; W/cm^2^: watt/centimetre square; mm: millimetre.

**Device** **Information**	Manufacturer	THOR Photomedicine Ltd.
	Model identifier	LX2.1
	Semi-conductor materials (emitter type)	GaAIAs
	Probe design	Single probe
	Beam delivery system	Hand-held probe
	Medical/laser class	3B laser
	NOHD	64 cm
	Laser-aiming beam	None
**Irradiation** **Parameters**	Wavelength	810 nm
	Operating emission mode	Continuous wave
	Beam profile	Gaussian distribution
**Treatment** **Parameters**	1/e^2^ Spot area size area	0.088 cm^2^
	1/e^2^ Spot size and shape	0.335 cm, circular
	Beam divergence full angle	10° × 54°
	Polarisation	Linear
	Therapeutic power output	200 mW
	Irradiance	2 W/cm^2^
	Irradiance at aperture (mW/cm^2^)	1.97 W/cm^2^
	Fluence (dose)	59.1 J/cm^2^
	Energy	6 J/point
	Total energy	54 J
	Power density (irradiance)	1.97 W/cm^2^
	Exposure time	30 s/point
	Time interval	Relatively alternate day, excluding weekend
	Treatment frequency	Twice a week (Mondays and Wednesdays)
	Total number of treatments	10
	Duration of treatment	5 consecutive weeks
	Number of irradiated points	9 points
	Irradiated target	Trigger points and sites of injury (affected areas)
	Scanning technique	Spot technique
	Light-tissue distance	<1 mm distance (non-contact)

**Table 3:** Shows the pharmacological treatment modality and doses over different time points. It shows that the doses of analgesic medications needed to be increased for all ten patients at the 3-month (T4) follow-up evaluation and 2/10 patients had another medication added. This indicates that for those patients recruited to the MED group, the initial doses of the medications had become ineffective to reduce the pain intensity below a tolerable level between 1–3 months after prescription. Abbreviations: BMS: burning mouth syndrome; OIN: oral iatrogenic neuropathy; Pt. no.: number of patients, mg: milligram.

Pt. No.	Condition	Prescribed Medication	Dose of the Medications and Follow-Up Time Points				
			Baseline (T0)	1-Month Follow-Up (T3)	3-Month Follow-Up (T4)	6-Month Follow-Up (T5)	9-Month Follow-Up (T6)
3	BMS	Nortriptyline	10 mg	10 mg	20 mg	20 mg	20 mg
2	OIN, BMS	Nortriptyline	20 mg	20 mg	40 mg	40 mg	40 mg
1	OIN	Nortriptyline Pregabalin	10 mg 100 mg	10 mg 100 mg	20 mg 200 mg	20 mg 200 mg	20 mg 200 mg
1	BMS	Pregabalin	100 mg	100 mg	200 mg	200 mg	200 mg
1	BMS	Pregabalin	25 mg	25 mg	50 mg	50 mg	50 mg
2	BMS	Gabapentin Nortriptyline	600 mg	600 mg	600 mg 10 mg	600 mg 10 mg	600 mg 10 mg

**Table 4:** Shows the baseline characteristics comparisons between the test (PBM) and pharmacotherapy comparison (MED) groups * Based on unpaired *t*-test (age), Fisher’s exact contingency analysis test (gender, type of NP and medical history) or non-parametric Mann–Whitney *U* ranks test (Onset).

Baseline Characteristics	PBM (*n* = 18)	MED (*n* = 10)	Different * (Y/N)	*p*-Value *
Age (years; mean ± SD)	58.00 ± 10.39	56.80 ± 10.84	N	0.7752
Gender (% female)	83.33 %	100.00%	N	0.5330
Type of NP (% BMS)	61.11 %	80.00%	N	0.4170
Onset (months; mean ± SD)	57.50 ± 47.93	38.90 ± 15.77	N	0.7500
Medical history (% fit and well)	61.11 %	90.00%	N	0.1937

## Data Availability

The data is contained within the article.

## Supplementary Materials

The following are available online at https://www.mdpi.com/article/10.3390/antiox11030533/s1, Supplementary File S1: CONSORT 2010 checklist of information to include when reporting a pilot or feasibility trial; Supplementary File S2: List of abbreviations.
