# Supplementary material for: Outpatient Oral Neuropathic Pain Management with Photobiomodulation Therapy: A Prospective Analgesic Pharmacotherapy-Paralleled Feasibility Trial
Source: Antioxidants (Basel). 2022 Mar 10;11(3):533. doi: 10.3390/antiox11030533 (PMC8944471; doi:10.3390/antiox11030533)
Supplement: Supplementary file 1 [file antioxidants-11-00533-s001.zip › Supplementary File S2.pdf]

Supplementary File S2: List of abbreviations (In alphabetical order)

Sr.no.	Abbreviation	Full form
1.	ATP	Adenosine triphosphate
2.	ANOVA	
3.	b-FGF	Basic fibroblast growth factor
4.	BMS	Burning mouth syndrome
5.	cAMP	Cyclic adenosine 3′,5′-monophosphate
6.	COX- 2	Cyclooxygenase-2
7.	CCO	Cytochrome C oxidase
8.	EQ-5D-5L	European quality of life-5 dimentions-5 levels
9.	EQ VAS	Euro QoL- visual analogue scale
10.	GAD-7	Generalized Anxiety Disorder 7-item scale
11.	HANDS	Hospital anxiety and depression scale
12.	OIN	Oral iatrogenic neuropathy
13.	OINI	Oral iatrogenic nerve injury
14.	IASP	International Association of Study Pain.
15.	ICHD	International Classification of Headache Disorders
16.	IL- 1β	Interleukin-1 beta
17.	IL-6	Interluekin-6
18.	IF-γ	Interferon- gamma
19.	IMMPACT II	Initiative on Methods, Measurement, and Pain Assessment in Clinical Trials
20.	J	Joule
21.	J/cm^2	Joules per square centimeter
22.	IFBMS	Local factors burning mouth syndrome
23.	LLLT	Low level laser therapy
24.	μm	Micrometer
25.	mJ	Millijoule
26.	mm	Millimetre
27.	mW	Milliwatt
28.	NGF	Nerve growth factor
29.	NP	Neuropathic pain
30.	McGill PQ,	McGill pain questionnaires
31.	MED	Pharmacotherapy group
32.	Min/s	Minute/s
33.	mRNA	Messenger Ribonucleic acid
34.	nm	Nanometer
35.	NO	Nitric oxide
36.	OHIP-14	Oral Health Impacts Profile
37.	PBM	Photobiomodulation
38.	PBM group	Photobiomodulation therapy group
40.	PGE 2	Prostaglandin E2
41.	PGI-I	Patients Global Impression of Improvement
42.	PIS_{max}	Pain intensity score (maximum)
43.	PI	Pain intensity
44.	PPI	Pressure pain intensity
45.	PROMs	Patient reported outcome measure
46.	QoL	Quality of life
47.	ROS	Reactive oxidase species

48.	RCT	Randomised clinical trials
49.	SAD	Social Anxiety Disorder
50.	SD	Standard deviation
51.	sec/s	Second/s
52.	SFBMS	Systemic factors burning mouth syndrome
53.	SF-36	Short-Form Health Survey
54.	SCL-90-R	Psychometric Symptom Checklist 90-R
55.	T0	Pre-treatment (Baseline)
56.	T1	Mid-treatment (end 5th session)
57.	T2	End-treatment (end 10th session)
58.	T3	One-month follow-up
59.	T4	Three-month follow-up
60.	T5	Six-months follow-up
61.	T6	Nine-months follow-up
62.	TGF-ß1	Transforming growth factor beta
63.	TNF-α	Tumor necrosis factor- alpha
64.	*t*-test	
64.	TN	Trigeminal nerve
65.	TRPV1	selective transient receptor potential vanilloid 1
66.	VAS	Visual analogue scale
67.	VEGF	Vascular endothelial growth factor
68.	W	Watt
69.	W/cm^2	Watt per quare centimetre
70.	%	percentage
